# Anti-Inflammatory Action of Angiotensin 1-7 in Experimental Colitis

**DOI:** 10.1371/journal.pone.0150861

**Published:** 2016-03-10

**Authors:** Maitham A. Khajah, Maryam M. Fateel, Kethireddy V. Ananthalakshmi, Yunus A. Luqmani

**Affiliations:** Faculty of Pharmacy, Kuwait University, PO Box 24923, Safat, 13110, Kuwait; Max-Delbrück Center for Molecular Medicine (MDC), GERMANY

## Abstract

**Background:**

There is evidence to support a role for angiotensin (Ang) 1–7 in reducing the activity of inflammatory signaling molecules such as MAPK, PKC and SRC. Enhanced angiotensin converting enzyme 2 (ACE2) expression has been observed in patients with inflammatory bowel disease (IBD) suggesting a role in its pathogenesis, prompting this study.

**Methods:**

The colonic expression/activity profile of ACE2, Ang 1–7, MAS1-receptor (MAS1-R), MAPK family and Akt were determined by western blot and immunofluorescence. The effect of either exogenous administration of Ang 1–7 or pharmacological inhibition of its function (by A779 treatment) was determined using the mouse dextran sulfate sodium model.

**Results:**

Enhanced colonic expression of ACE2, Ang1-7 and MAS1*-R* was observed post-colitis induction. Daily Ang 1–7 treatment (0.01–0.06 mg/kg) resulted in significant amelioration of DSS-induced colitis. In contrast, daily administration of A779 significantly worsened features of colitis. Colitis-associated phosphorylation of p38, ERK1/2 and Akt was reduced by Ang 1–7 treatment.

**Conclusion:**

Our results indicate important anti-inflammatory actions of Ang 1–7 in the pathogenesis of IBD, which may provide a future therapeutic strategy to control the disease progression.

## Introduction

The renin-angiotensin-aldosterone system (RAAS) plays an important role in the homeostatic control of cardiovascular, renal and adrenal functions. This peptide-based hormonal system is comprised of two main pathways; angiotensin converting enzyme/angiotensin II/angiotensin type 1/2 receptor (ACE/Ang II/AT1/2R; which is the classical RAAS), and ACE2/Ang 1-7/MAS-1 receptor (MAS1-R) (a recently described version of RAAS) [1, 2]. The proteolytic enzyme renin is released by the juxtaglomerular cells in the kidneys to cleave the N-terminal region of angiotensinogen to form Ang I (or decapeptide). Ang I is further degraded to Ang II by the removal of two C-terminal amino acids by proteases such as ACE and chymas-1. Ang II binds to two main receptors; AT1R and AT2R. Whereas AT1R plays an important role in blood pressure control and cardiac cell remodeling, AT2R antagonizes the signaling associated with AT1R stimulation [1]. Furthermore, Ang II can be converted to smaller peptide products with various biological activities. One of these, Ang 1–7, can be synthesized not only from Ang II (via postproline carboxypeptidase) but also from Ang I (via tissue endopeptidases; neprilysin, prolyl endopeptidase, and thimet oligopeptidase), or directly from Ang 1–9 following its formation from Ang 1–10 by ACE2, bypassing the synthesis of Ang II [3]. Ang 1–7 induces its biological effects through the only known receptor for this peptide; the MAS-1 R oncogene G-protein coupled receptor [4]. The physiological roles of Ang 1–7 on renal function, fluid homeostasis, vascular tone and cardiac contractility and remodeling are well documented, and most of these actions are directly opposed to Ang II-mediated effects [5–8]. It is thought that the beneficial effects of ACE-inhibitors (ACEIs) and angiotensin receptor blockers (ARBs) on blood pressure control and in delaying/inhibiting the cardiac remodeling process is through increasing serum levels of Ang1-7 [9–12].

Several studies have suggested an important role for Ang II in the pathogenesis of inflammatory bowel disease (IBD), a chronic disorder of the gastrointestinal tract which comprises two conditions: Crohn’s disease and ulcerative colitis [13, 14]. When the production and/or the activity of Ang II is reduced, colitis severity is ameliorated in various animal models of IBD through various mechanisms. Treatment with ACEIs or ARBs resulted in significant reduction in colitis severity in 2, 4, 6-trinitrobenzene sulphonic acid (TNBS) and dextran sulfate sodium (DSS) induced models of experimental colitis [15–21]. Furthermore, colitis severity can also be ameliorated by making mice homozygous for targeted disruption of the angiotensin gene [21] or ATR1a deficiency [22]. These protective effects are considered to be due in part to inhibition of Ang II mediated enhancement of NFκB phosphorylation, adhesion molecule expression in the gut (the mucosal addressin; MAdCAM-1) and serum pro-inflammatory cytokine release (TNFα, IFNγ, IL-1β). Furthermore, ACEI or ARB treatment in mice results in reduced colonic epithelial cell apoptosis and enhanced expression of the anti-inflammatory cytokine IL-10. Interestingly, ACE gene polymorphism was detected in IBD patients and is associated with reduced ACE serum levels; this might be associated with the disease pathogenesis and its extra-intestinal side effects [23–25]. It is thought that chronic treatment with RAAS blockers (ACEI or ARBs) may lead to increased Ang 1–7, which could then oppose the harmful effects of Ang II in the cardiac and renal system, and possibly in the gut as well.

Anti-inflammatory effects of Ang 1–7 have been reported in atherosclerosis plaque [26] and in an arthritic model [27] which was mediated through inhibition of NFκB activity and reduction of cytokines and chemokines such as TNFα, IL-1β, MCP-1 and CXCL1. Several reports [28–31] have suggested that Ang 1–7 treatment reduces the activity of intracellular signaling molecules such as MAPK family (p38, ERK1/2 and JNK), protein kinase C (PKC) and c-SRC kinase, which play an important role in intensifying the inflammatory response. Ang 1–7 has been found to reduce the severity of allergic inflammation in mice by suppressing the activity of ERK1/2 and NFκB pathways [32]. Of particular interest, ACE2 (the main enzyme responsible for Ang 1–7 generation) expression has been observed in epithelial and sub-mucosal cells throughout the gut, with significant expression in the ileum and the colon [33–35].

In the present study, we used the mouse DSS colitis model to examine the ability of Ang 1–7 to influence colitis severity. We show for the first time that the expression of ACE2/Ang 1-7/MAS-1 R are modulated post colitis induction. Blockade of the endogenous function of Ang 1–7 (by the MAS-1 R antagonist A779) exacerbated colitis severity, while daily administration of Ang 1–7 ameliorated it. The anti-inflammatory effects of Ang 1–7 were associated with reduction in the expression level of Ang II and the phosphorylation of p38 MAPK, ERK1/2 and Akt in the colon.

## Materials and Methods

### Animals

Male and female BALB/c mice (1:1 ratio, 6–10 weeks old, mean weight 20 g.) were supplied by the Animal Resource Center of the Health Sciences Center at Kuwait University. All animals were kept under standard conditions including controlled temperature (25°C), a 12-h light-dark cycle and had free access to food and drinking water *ad libitum*. All experimentations were approved by the Animal Care Committee at Kuwait University Health Sciences Center and conformed to their rules and regulations. During this study, the general health and wellbeing of the mice was continuously monitored and overseen by a veterinary physician in the animal house. All mice were monitored for their eating/drinking habits, activity, or other severe signs of hunched or lateral recumbency or starry fur or lethargy. There were no signs of illness or mortality during the treatment time points that required sacrificing the animals.

The number of animals in each control and treated group for each set of experiments is indicated in the respective figure/table legends.

### Induction of colitis

Colitis was induced in mice by delivering DSS polymers (3.5% w/v, m.wt 40 kD; MP Biomedicals, France) in autoclaved drinking water and provided *ad libitum* for 4–7 days [36]. Control (untreated; UT) mice received autoclaved tap water only. The DSS solution was replaced every 2 days for the duration of the experiment. There was no difference in the amount of water consumption by mice between the groups. Decrease in body weight provides indirect indication of colitis severity. Mice were sacrificed by cervical dislocation at 4–7 days post-colitis induction. Blood samples were collected by cardiac puncture using a heparinized syringe (for total and differential WBC count). Colons were resected and fixed in 10% formalin solution.

#### Treatment protocols

Angiotensin fragment 1–7 acetate salt hydrate (Ang 1–7; m.wt 899; Sigma-Aldrich, St Louis, USA) was dissolved in 0.9% saline (vehicle) at 1 mg/ml and stored at -80°C. Various doses (0.01, 0.06, 0.1, 0.3 and 1 mg/kg) were freshly prepared from the stock each day of the experiment, and administered to mice by daily intra-peritoneal (i.p) injections in a volume of 500 μl per injection, either before (prophylactic approach) or after (treatment approach) DSS treatment. A779 (MAS-1 R antagonist; m.wt 873; GenScript, USA) was similarly dissolved in distilled water at 1 mg/ml and stored at -80°C. A freshly prepared dose of 1 mg/kg was administered to a second group of mice by daily i.p injections in a volume of 500 μl daily (for 4 days) along with colitis induction (prophylactic approach). A third group of mice received DSS containing water and daily i.p injections of 0.9% saline (vehicle). The fourth group received DSS containing water along with daily i.p injections with dexamethasone (DEX) at doses of 0.01–1.0 mg/kg or its vehicle (0.9% saline) (prophylactic approach).

#### Gross (macroscopic) assessment of colitis severity

Using sterile forceps and scissors, the entire colon of each mouse was removed by a ventral midline incision and opened longitudinally. Its length and maximal bowel thickness was measured (in mm) with calipers. Several other macroscopical parameters were used to assess the colitis severity including stool consistency, blood in stool, adhesion, erythema, edema and ano-rectal bleeding [36]. The data is presented as the percentage of mice in each group showing these features.

#### Histological (microscopic) assessment of colitis severity

The colon was cleaned from stools and blood with a few drops of sterile 0.9% saline and ‘swiss-rolled’ from the descending to the ascending part. Samples were fixed in 10% neutral buffered formalin and placed in tissue processing and embedding cassettes in PBS for a few minutes and then overnight in a LEICA ASP 3005 tissue processing machine. Tissues were rinsed in two changes of formalin, then dehydrated in several changes of graded alcohol (70%, 90% and 100%). Three changes of xylene were used for tissue condensation and clearing. Using an SLEE-MPS embedding machine, processed tissues were embedded in paraffin wax (24 h at room temperature) and stored at 4°C before trimming and sectioning using an LEICA RM 2235 microtome. Sections (6 μm thick) were floated in a water bath and then placed on uncoated slides at 37°C overnight. After de-paraffinisation in three changes of xylene (5 min each) and rehydration by serial immersion for 2–3 min in each of absolute, 90% and 70% alcohol, sections were washed briefly with distilled water and stained in Meyer's alum haematoxylin solution for 7 min followed by thorough rinsing with running tap water. Before counter-staining sections in eosin solution for 2 min, slides were dehydrated in graded alcohol. A clearing step was performed by rinsing in three changes of xylene (2 min each) followed by mounting with DPX.

The stained sections were (blindly) scored by 3 observers using a standard semi-quantitative histology scoring system [36–38] which graded the following features: extent of destruction of normal mucosal architecture (0, normal; 1, 2, and 3, mild, moderate, and extensive damage respectively), presence and degree of cellular infiltration (0, normal; 1, 2, and 3, mild, moderate, and transmural infiltration respectively), extent of muscle thickening (0, normal; 1, 2, and 3, mild, moderate, and extensive thickening respectively), presence or absence of crypt abscesses (0, absent; 1, present) and the presence or absence of goblet cell depletion (0, absent; 1, present). The scores for each feature were summed with a maximum possible score of 11. The extent of ulceration was determined on each section along the muscularis mucosa and expressed as percentage ulcerated mucosa [38].

#### Differential white blood cell counts

During dissection, blood was collected by cardiac puncture using a 1 ml heparinized syringe. To determine differential white blood cell count (WBC), a blood smear was made on a glass slide by placing a drop of blood on one slide surface and using a second glass slide to push the blood forward from the edge of the drop, and left to dry for 24h. SIEMENS, Diff-Quik™ Stain Set was used for rapid differential staining. Slides were dipped 5 times in a methanolic fixative to stabilize cellular components, then dipped 10 times in a buffered solution of eosin Y and 7 times in a buffered solution of thiazine dye (methylene blue and azure A). Finally, slides were rinsed in distilled water, dried and stored at room temperature. WBCs were identified as monocytes, neutrophils, basophils, eosinophils and lymphocytes. A total of 100 cells were randomly counted in each slide and the data are presented as % of the presence of each cell type.

### Western blotting

Colon tissue samples (descending part) were cut and homogenized (with Teflon glass homogenizer) in 1 ml of buffer composed of 1.2g of 50 mM HEPES, 0.3g of 50 mM NaCl, 1ml of 0.5M EDTA, 1ml of 1% Triton X-100 and 98ml of deionized water. A protease inhibitor cocktail (10μg/ml aprotinin, 10μg/ml leupeptin and 100μM PMSF) was added separately. Homogenates were centrifuged at 1,800 rpm for 10 min at 4°C, and the supernatant collected. Protein concentration was determined by the Bradford assay (Bio-Rad, Hercules, CA). Samples containing 50 μg protein were dissolved in an equal volume of 2 x Lammeli sample buffer and β-mercaptoethanol, heated at 90°C for 10 min and loaded onto a 12.5% SDS-polyacrylamide gel and electrophoresed at 125 V for 1 h. Proteins were transferred (at 100 V for 1 h) onto a PVDF membrane (Millipore, Ireland) and then blocked with 4% BSA for 90 min before overnight incubation at 4°C with primary antibodies for actin (Cell Signaling Technology, Boston, MA, USA; 1/1000 dilution), MAS-1 R (M 13, Santa Cruz, TX, USA; 1/100 dilution), Ang II (Novus Biological; 1/500 dilution), and ACE2 (Santa Cruz, TX, USA; 1/50 dilution). Membranes were washed 3 times for 1 h with 1x TBS-T buffer and incubated with appropriate horseradish peroxidase (HRP)-labeled secondary antibodies [Anti-rabbit IgG, HRP linked antibody (Cell Signaling Technology, Boston, MA, USA; 1/1000 dilution), and Donkey anti-goat IgG- HRP linked antibody (Santa Cruz, TX, USA; 1/100 dilution)]. Bands were visualized using Super Signal ECL substrate (Thermo Scientific, Rockford, USA) and Kodak X-ray film.

### Immunofluorescence

Colon sections (5μm) were deparaffinized, rehydrated through a series of washes in graded ethanol and water, followed by an antigen retrieval step (by boiling the sections in 10 mM sodium citrate buffer, pH 6.0 for 20 min). Sections were then incubated in blocking solution (5% bovine serum albumin (BSA) + 0.3% Triton X-100 in PBS) for 1 h, followed by incubation overnight at 4°C with primary antibodies [p-ERK1/2, p-Akt (1:50 dilution), p-p38 MAPK and MAS-1R (1:100 dilution); Cell Signaling, USA, and Ang II (1/50 dilution); Novus Biological] diluted in 1% blocking solution. On the following day, sections were washed and incubated with secondary antibody conjugated to Alexa Fluor 555 (Goat anti rabbit SFX kit; Life Technologies,USA, 1:400 dilution) for 2 h at room temperature) in the dark. After washes in PBS sections were stained with 4’,6 diamidino-2- phenylindole and mounted. Images were captured on a ZIESS LSM 700 confocal microscope and fluorescence intensity estimated in defined fields using Image J software package.

### Ang 1–7 protein measurement in plasma and colon homogenates

Colon tissue (100 mg/ml) was homogenized as described above and sonicated for 1 min for further disruption of the cell membrane. Homogenates were centrifuged for 15 min at 5,000 rpm and the supernatants collected for subsequent analysis. Blood was collected by cardiac puncture, centrifuged at 5,000 rpm for 10 min and the plasma stored at– 80°C.

Ang 1–7 was measured using Mouse Angiotensin 1–7 Elisa Kit (MyBiosource # MBS700453). following the manufacturer’s protocol: 50 μl of standards, samples and blank (PBS) were pipetted into the pre-coated wells and mixed with 5 μl of the balance solution. Enzyme conjugate (50 μl) was added to samples (except blank) and kept for 1 h at 37°C in the dark. After removal of suspension, wells were washed five times with 1 x wash solution prior to addition of HRP substrates A and B and 15 min later, of stop solution. Intensity of the yellow complex was measured at 450 nm and concentration of Ang 1–7 interpolated from a standard curve.

### Statistical analysis

Data were analyzed using GraphPad Prism version 5.0 for Windows (GraphPad Software, Calfornia, USA). Differences between groups were assessed using unpaired Students' t test or one-way ANOVA, with p ≤ 0.05 being regarded as significant.

## Results

### Effect of DSS treatment on Ang 1–7 levels

**Fig 1:**
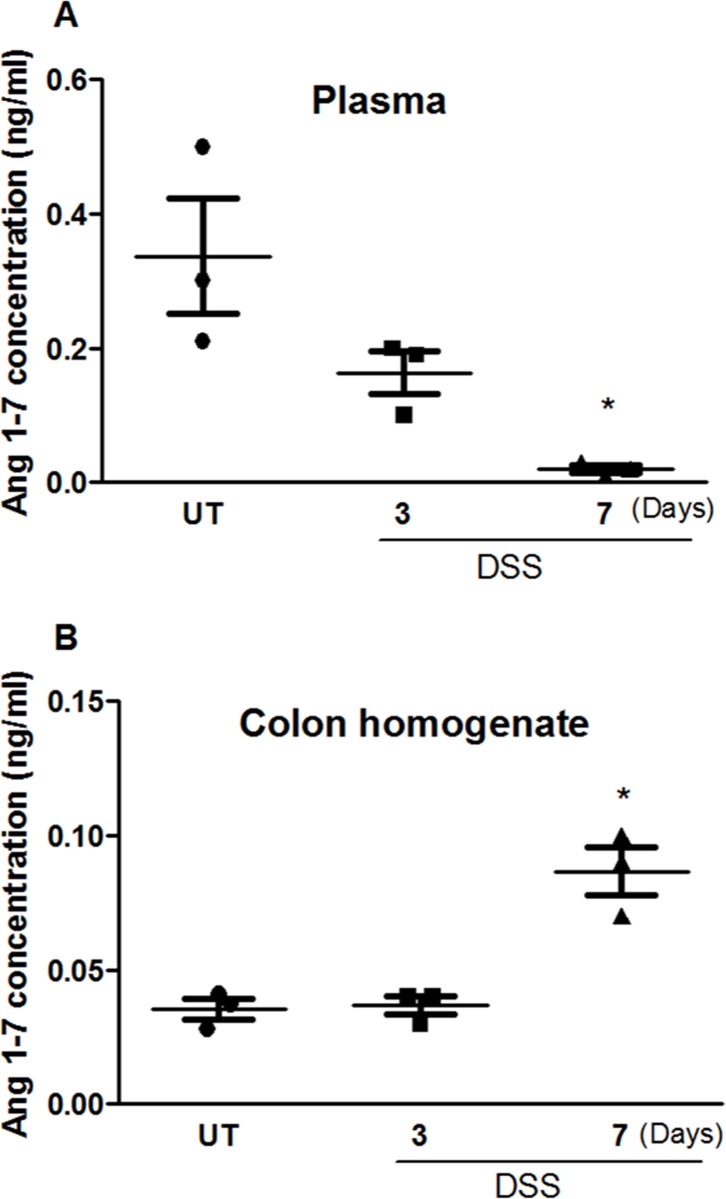
Ang 1–7 protein levels post-colitis induction. Ang 1–7 levels were measured in plasma (A) and colon homogenates (B) at 3 and 7 days after treatment with 3.5% DSS (open bars), and compared to untreated (UT) mice (solid bars). Histobars represent the mean values ± SEM for 3 mice per group. Asterisks indicate significant difference from UT group with p<0.05.

A seven fold decrease in the plasma level of Ang 1–7 was demonstrated in DSS treated mice compared to untreated (UT) group at day 7 post colitis induction (Fig 1A). On the other hand, a significant increase in Ang 1–7 was observed in colon homogenates of DSS treated mice at day 7 (0.09 ng/ml) compared to UT mice (0.04 ng/ml) as shown in Fig 1B.

### Effect of DSS treatment on colonic expression of Ang II, ACE2 and MAS-1 R

**Fig 2:**
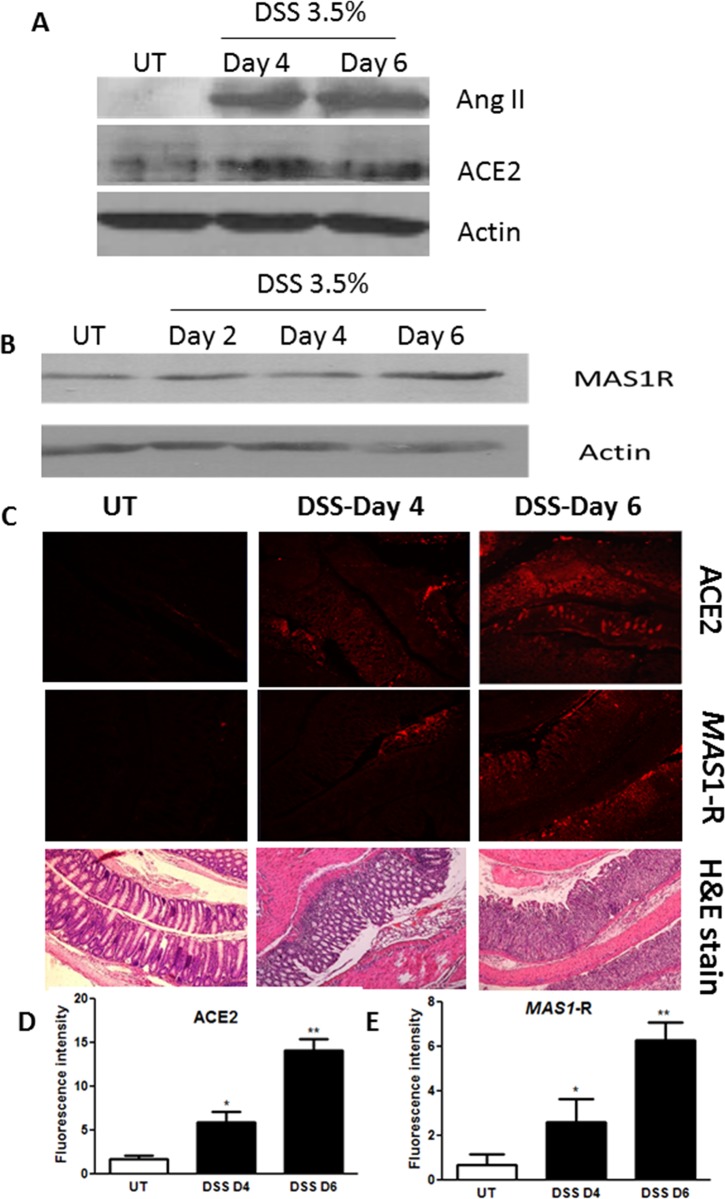
Effect of DSS treatment on the levels of the RAAS components. Colonic protein levels of Ang II and ACE2 (A), and MAS-1 R (B) were determined by western blotting at various times post colitis induction with DSS (blots represent one of 3 similar experiments). Panel C shows immunofluorescence staining of ACE2 and MAS-1 R in untreated mice (UT) and at days 4 and 6 post colitis induction, predominantly in the mucosal layers, as evidenced from the H&E staining. Histobars in panels D and E represent the mean (± SEM) fluorescence intensity for 3 mice per group for ACE2 and MAS-1 R respectively. Asterisks indicate significant difference from UT group with p<0.05 (*) and p<0.001 (**).

Ang II expression determined by immunoblotting was increased at days 4 and 6 post-colitis induction compared to UT mice (Fig 2A), as was ACE2. An elevation was seen at day 6 in the MAS-1 R (Fig 2B). Immunofluorescent staining showed presence of ACE2 and MAS-1 R in the mucosal layers of colon sections (Fig 2C) observed at days 4 and 6 post-colitis induction, whereas very little reaction was detectable in colon tissue of untreated control mice.

### Effect of Ang 1–7 treatment on colitis severity

**Fig 3:**
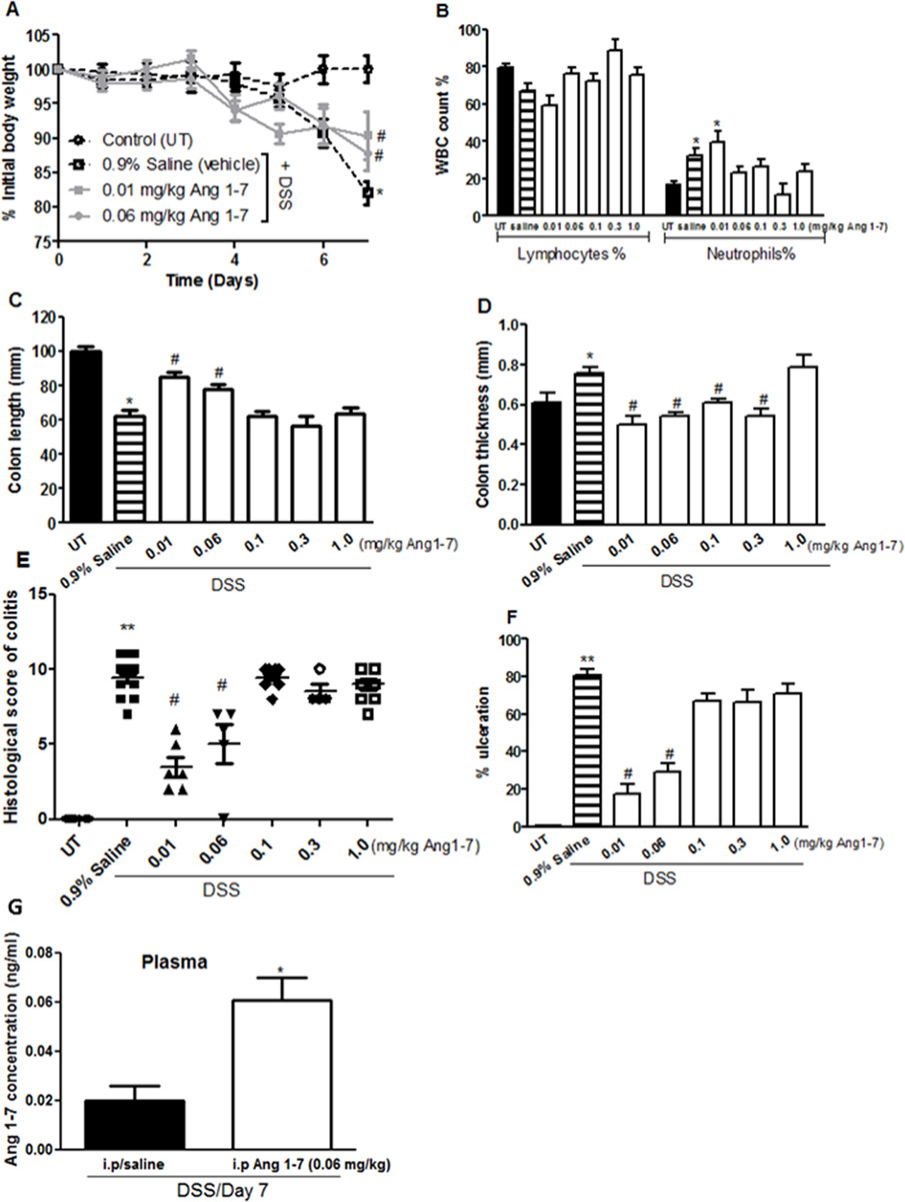
Effect of Ang 1–7 treatment on colitis severity. Panel A shows % body weight changes in saline (hatched line, open squares) or Ang 1–7 treated mice (gray lines) after DSS administration compared to untreated (UT) mice receiving tap water only (hatched line, open circles). Panel B shows % of circulating lymphocytes and neutrophils determined at day 7 post colitis induction in saline (hatched bars) or Ang1-7 treated mice (open bars) compared to UT group (solid bars). Colon length (panel C) and thickness (panel D) was determined in saline (hatched bars) or Ang1-7 treated mice (open bars) compared to UT group (solid bars). Panels E and F represent histological assessment of colitis severity and the % of ulceration in the whole colon section respectively in the groups indicated. Panel G represent plasma levels (ng/ml) of Ang 1–7 in mice treated with DSS (for 7 days) plus either daily i.p saline or Ang 1–7 (0.06 mg/kg). Histobars represent means ± SEM for 4–18 mice in each group (8 for UT, 18 for DSS/i.p saline, 6 for Ang 0.01 mg/kg, 5 for Ang 0.06 mg/kg, 9 for Ang 0.1 mg/kg, 4 for Ang 0.3 mg/kg and 9 for Ang 1.0 mg/kg). Asterisks denote significant difference from UT mice with p<0.05 (*) and p<0.001 (**). # denotes significant difference from DSS/i.p saline treated mice with p<0.05.

The effect of Ang 1–7 on modulating colitis severity in mice was tested by daily i.p injections of various doses of the peptide. DSS administration resulted in approximately 20% drop in body weight from day 5 onwards (in contrast with mice receiving tap water only). Ang 1–7 at doses of 0.01 and 0.06 mg/kg (but not at higher doses of 0.1–1 mg/kg; data not shown) reduced this drop in body weight by 5–10% compared with the DSS/saline i.p treated group (Fig 3A). DSS treatment in the DSS/saline i.p group resulted in increased circulating neutrophils; this was prevented by Ang 1–7 treatment at doses of 0.06–1 mg/kg (Fig 3B). No significant changes in circulating lymphocytes were observed between control and treatment groups. The decrease in colon length and thickness seen after DSS treatment was prevented by Ang 1–7 treatment at doses of 0.01–0.06 mg/kg but not at higher doses (Fig 3C and 3D).

**Fig 4:**
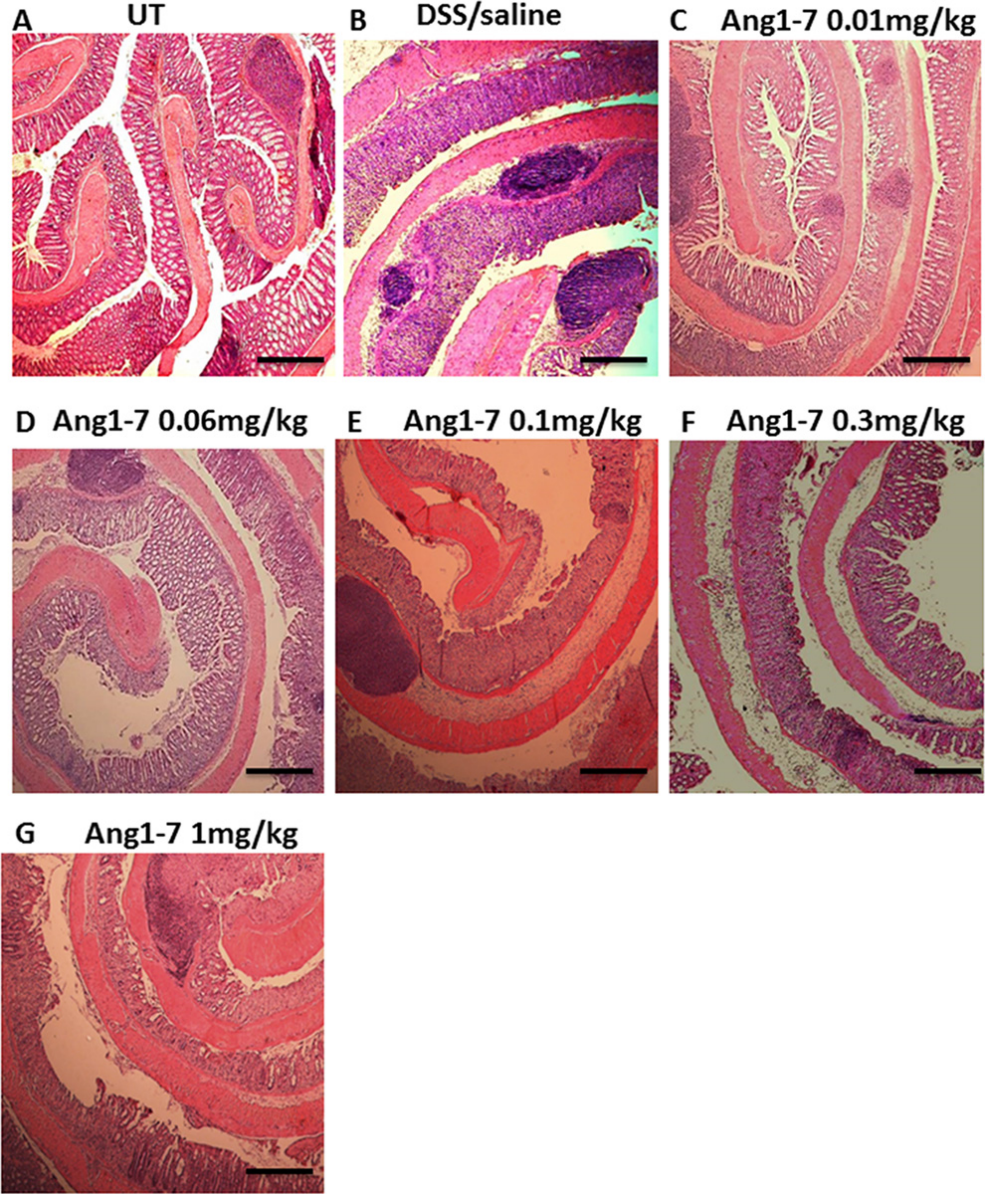
H&E stained colon sections of mice treated with Ang 1–7 along with colitis induction by DSS. Panel A illustrates a colon section taken from UT mouse showing normal regular mucosal architecture. Panel B illustrates a colon section taken from mouse treated by daily i.p saline plus DSS where there is significant mucosal destruction and immune cells recruitment. Panels C-G illustrate typical colon sections taken from mice treated with Ang1-7 plus DSS. Treatment with 0.01–0.06 mg/kg doses improved mucosal integrity and reduced the enhanced immune cell recruitment (panels C and D). This anti-inflammatory effect of Ang 1–7 was lost with higher doses (panels E-G). Bars represent 150μm.

As indicated in Table 1, at the gross level, DSS administration for 7 days with daily i.p injections of saline (vehicle) resulted in diarrhea, blood in stool, adhesion, erythema, edema and ano-rectal bleeding, none of which was seen in the UT group. Daily administration of Ang 1–7 at doses of 0.01–0.06 mg/kg significantly reduced most of these effects. The presence of erythema was reduced by 40–60%, incidence of diarrhea by 30–60%, ano-rectal bleeding by 6–50%, blood in stool by 30%, and edema by 20–30% relative to DSS/saline i.p group. Ang 1–7 at 0.1 mg/kg dose reduced ano-rectal bleeding, blood in stool and erythema by 15–40% but at 0.3–1.0 mg/kg, this protective effect was lost. The histological score of colitis correlated well with the macroscopic score (as shown in Fig 3E); a significant reduction in colitis severity was seen with Ang1-7 treatment only at the lower doses of 0.01–0.06 mg/kg. Similarly, the % of ulceration (Fig 3F) was significantly reduced (40–55%) but again only with the lower dose of 0.01–0.06 mg/kg Ang 1–7. Furthermore, daily i.p injections of Ang 1–7 at 0.06 mg/kg dose significantly increased the plasma levels of Ang 1–7 compared to mice receiving daily i.p saline at day 7 post-colitis induction (Fig 3G). The histological features of the resected tissues are illustrated in Fig 4. Compared to the normal architecture in untreated (UT) controls, DSS/i.p saline treatment for 7 days resulted in significant mucosal destruction and infiltration by immune cells (Fig 4B). Injection of DSS treated animals with 0.01–0.06 mg/kg doses of Ang 1–7 improved mucosal integrity and reduced the degree of immune cell recruitment to the colon (Fig 4C and 4D). This anti-inflammatory effect was lost with higher Ang 1–7 doses (Fig 4E, 4F and 4G).

**Table 1:** Effect of Ang1-7 on macroscopic features of colitis when administered simultaneously with DSS.

% of mice displaying each parameter						
Mice group	Diarrhea	Blood in stool	Adhesion	Erythema	Edema	Ano-rectal bleeding
UT	0	0	0	0	0	0
0.9% Saline + DSS	94	55	100	83	100	61
Ang 1–7 (0.01 mg/kg) + DSS	33*	22*	89*	22*	67*	55*
Ang 1–7 (0.06 mg/kg) + DSS	67*	22*	100	44*	78*	11*
Ang 1–7 (0.1 mg/kg) + DSS	91	36*	100	45*	91	45*
Ang 1–7 (0.3 mg/kg) + DSS	100	50	100	50*	100	50*
Ang 1–7 (1.0 mg/kg) + DSS	100	55	100	67*	89	67

Colitis severity (reflected by indicated parameters) was assessed in untreated (UT) mice or in mice receiving daily i.p saline or Ang 1–7 for 7 days along with DSS treatment. Numbers are the means of 4–18 mice for each group (8 for UT, 18 for DSS/i.p saline, 6 for Ang 0.01 mg/kg, 5 for Ang 0.06 mg/kg, 9 for Ang 0.1 mg/kg, 4 for Ang 0.3 mg/kg and 9 for Ang 1.0 mg/kg).

* denote significant difference from DSS/i.p saline treated mice.

### Effect of Ang 1–7 treatment on established colitis

**Fig 5:**
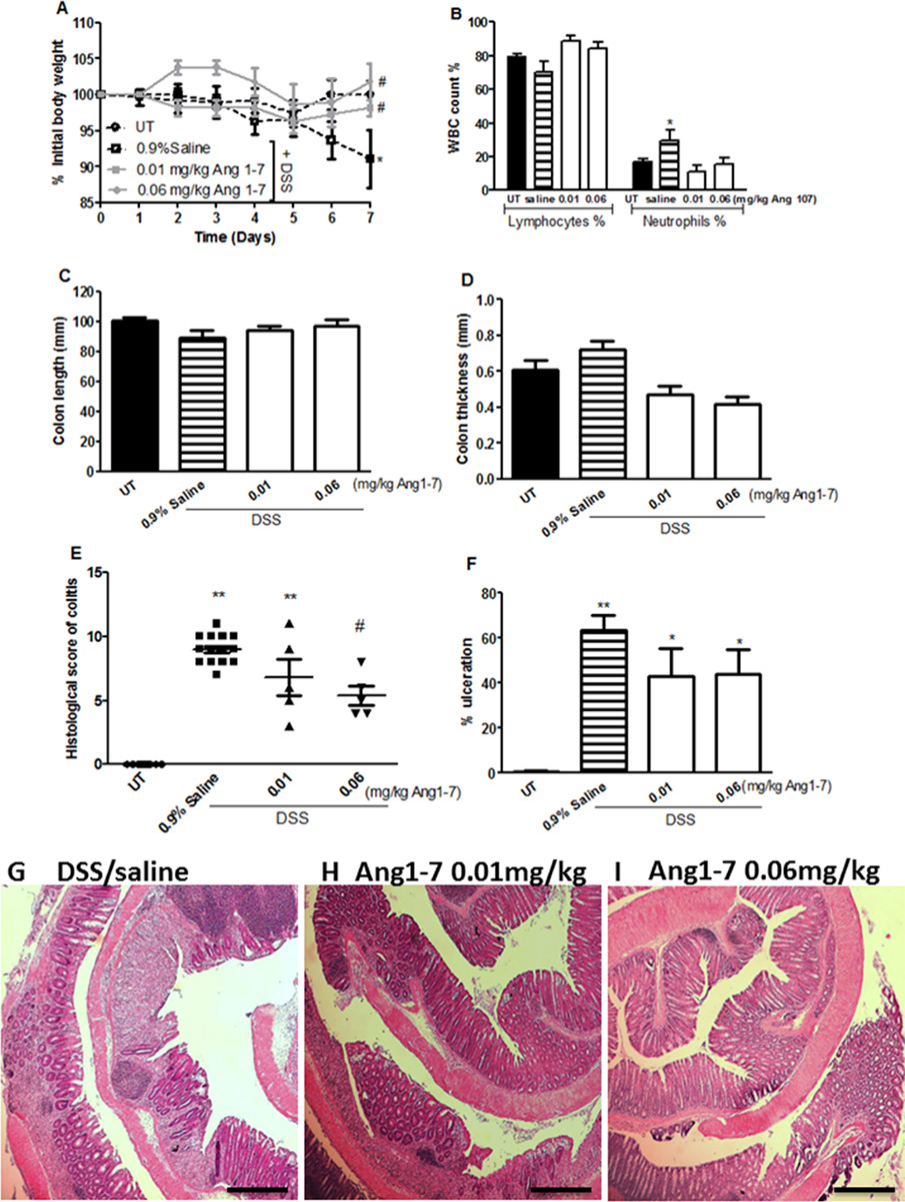
Effect of Ang 1–7 treatment on established colitis. Panel A shows % body weight change in mice injected daily (for 3 days) with saline (hatched line, open squares) or Ang 1–7 (gray lines) post-DSS administration (given for 4 days) compared to UT mice receiving tap water only throughout (hatched line, open circles). Panel B shows % of circulating lymphocytes and neutrophils. Colon length (panel C) and thickness (panel D) was determined in saline (hatched bars) or Ang1-7 treated DSS mice (open bars) compared to UT group (solid bars). Panels E and F represent quantitatively the histological assessment of colitis severity and the % of ulceration in the whole colon section for each group as indicated. Histobars represent means ± SEM for 5–18 mice in each group (9 for UT, 18 for DSS/i.p saline and 5 for Ang 0.01 mg/kg and Ang 0.06 mg/kg). In panels A asterisks denote significant difference from UT mice with p<0.05 (*) and p<0.001 (**). # denotes significant difference from DSS/ i.p saline treated mice with p<0.05. Panel G shows colon section taken from a mouse treated with DSS for 4 days followed by 3 days of i.p saline (vehicle) indicating the extent of mucosal destruction and immune cells recruitment. Panels H and I show colon sections taken from DSS mice subsequently treated with Ang1-7. Significant improvement in the mucosal integrity and reduction in immune cell recruitment can be seen. Bars represent 150μm.

To determine whether Ang 1–7 can reduce the severity of colitis once it is established, mice were given daily i.p injections of Ang 1–7 (for 3 days) 4 days after DSS administration (when effects are less severe). By 7 days, at both doses of 0.01 or 0.06 mg/kg, Ang 1–7 reversed the loss in body weight induced by DSS (Fig 5A). In addition, the elevation in % circulating neutrophils seen with DSS treatment was also reversed by Ang 1–7 back to levels seen in the UT group (Fig 5B). The small increase in colon thickness observed in the DSS group was reversed by Ang 1–7 though this effect did not reach statistical significance (Fig 5D). Histological examination (Fig 5G–5I and quantified in Fig 5E and 5F) showed that Ang 1–7 could reverse some of the DSS induced mucosal damage and ulceration, though in the case of the latter it did not reach statistical significance. At the macroscopic level, Ang 1–7 was seen to reduce the DSS induced increases in edema and erythema but not in adhesion (Table 2).

**Table 2:** Effect of Ang1-7 on macroscopic features of colitis when administered after DSS treatment.

% of mice displaying each parameter						
Mice group	Diarrhea	Blood in stool	Adhesion	Erythema	Edema	Ano-rectal bleeding
UT	0	0	0	0	0	0
0.9% Saline + DSS	0	0	100	80	100	0
Ang 1–7 (0.01 mg/kg) + DSS	0	0	100	0*	40*	0
Ang 1–7 (0.06 mg/kg) + DSS	0	0	100	60*	40*	0

Colitis severity was assessed by macroscopic examination in UT mice and in mice treated for 3 days with i.p saline or Ang 1–7 post DSS treatment (for 4 days). Numbers are the means of 5–18 mice for each group (9 for UT, 18 for DSS/i.p saline and 5 for Ang 0.01 mg/kg and Ang 0.06 mg/kg).

* denote significant difference from DSS/i.p saline treated mice.

### Effect of dexamethasone treatment on colitis severity

**Fig 6:**
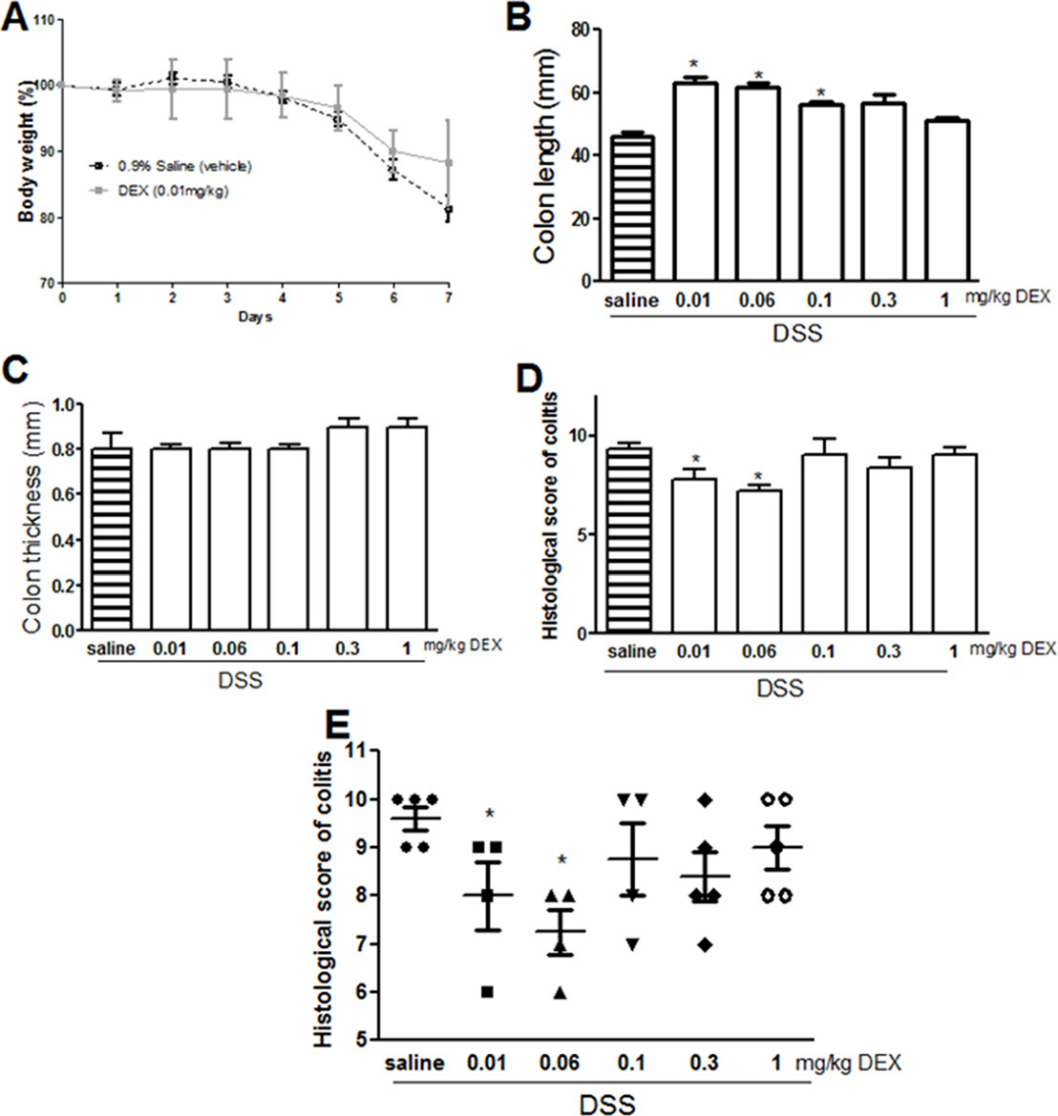
Effect of dexamethasone on colitis severity. Panel A shows % body weight change in saline (hatched line) or DEX treated (gray line) mice given DSS administration for 7 days. Panels B and C show colon length and thickness (in groups as indicated). Panels D and E represent histological assessment of colitis severity and the % of ulceration in the whole colon section respectively in the groups indicated. Histobars represent means ± SEM for 4 (DEX 0.01 mg/kg, DEX 0.06 mg/kg and DEX 0.1 mg/kg) or 5 (DSS/i.p saline, DEX 0.3 mg/kg and DEX 1.0 mg/kg) mice per group. Asterisks denote significant difference from DSS/ i.p saline treated mice with p<0.05.

We compared the effects of Ang 1–7 with the classic anti-inflammatory agent dexamethasone (DEX) using the same treatment approach. The DSS induced loss in body weight was partly reversed after 7 days of DEX treatment at 0.01 mg/kg dose (Fig 6A). As observed with Ang 1–7, this effect was lost at higher doses. DEX did not reverse the increase in neutrophils (data not shown). Whereas there was no difference in the colon thickness, colon length was significantly increased by DEX treatment at 0.01–0.1 mg/kg doses (but not higher) compared to DSS/i.p saline group (Fig 6B). Both histological score of colitis and extent of ulceration (Fig 6D and 6E) were reduced at low (but not higher) doses of DEX. With respect to the macroscopic parameters, DEX treatment specifically at 0.01 mg/kg dose significantly reduced diarrhea, blood in stool, ano-rectal bleeding and erythema, but did not have any effect on either the edema or adhesion (Table 3).

**Table 3:** Effect of DEX on macroscopic features of colitis when administered simultaneously with DSS.

% of mice displaying each parameter						
Mice group	Diarrhea	Blood in stool	Adhesion	Erythema	Edema	Ano-rectal bleeding
UT	0	0	0	0	0	0
0.9% Saline + DSS	100	100	100	100	100	86
DEX (0.01 mg/kg) + DSS	40*	40*	100	10*	100	40*
DEX (0.06 mg/kg) + DSS	100	20*	100	10*	100	100
DEX (0.1 mg/kg) + DSS	100	100	100	100	100	90
DEX (0.3 mg/kg) + DSS	100	100	100	100	100	60*
DEX (1.0 mg/kg) + DSS	100	100	100	100	100	80

Colitis severity was assessed by macroscopic examination in UT mice, or in mice treated with i.p saline or DEX for 7 days along with DSS treatment. Numbers are the means of 4 (DEX 0.01 mg/kg, DEX 0.06 mg/kg and DEX 0.1 mg/kg) or 5 (DSS/i.p saline, DEX 0.3 mg/kg and DEX 1.0 mg/kg) mice per group.

* denote significant difference from DSS/i.p saline treated mice.

### Effect of inhibiting endogenous Ang 1–7 levels on colitis severity

**Fig 7:**
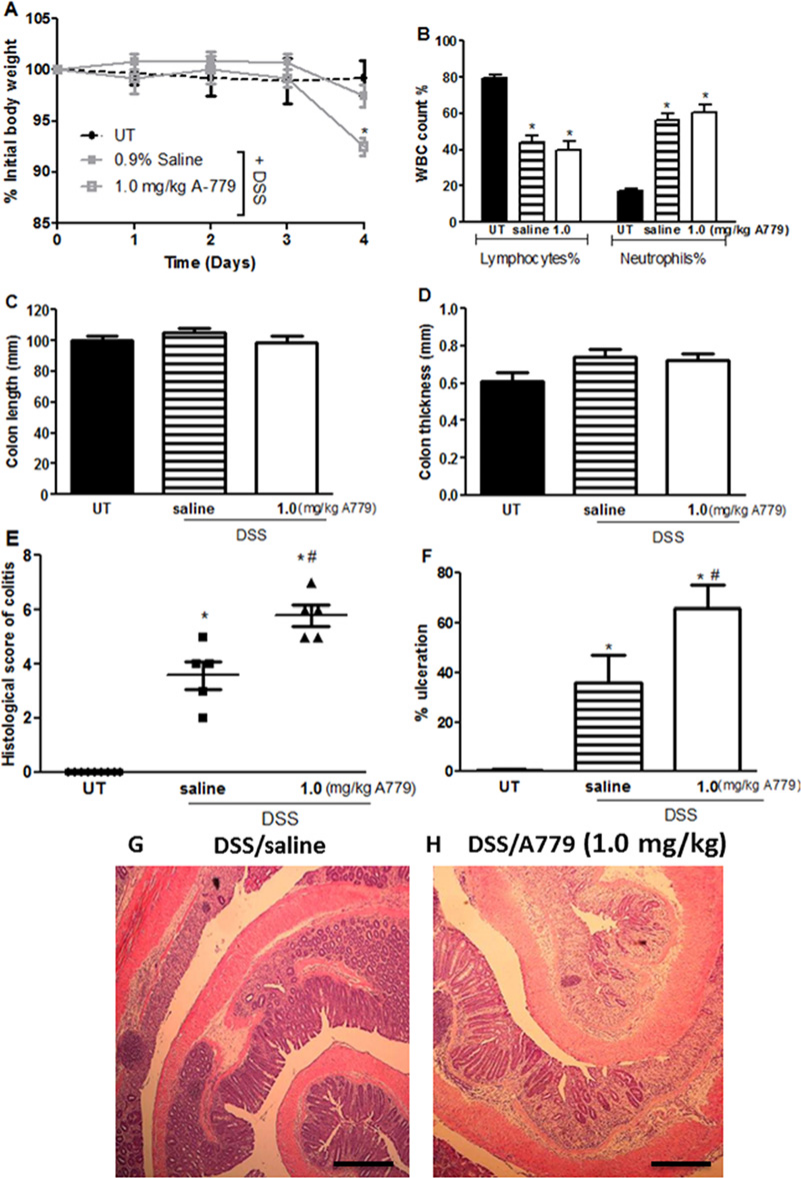
Effect of A779 on colitis severity. Panel A shows % body weight change in untreated (UT; hatched line, solid circles) or DSS treated (for 4 days) mice given daily i.p injections of A779 (gray line, open squares) or saline (gray line, solid squares). Panel B shows % of circulating lymphocytes and neutrophils determined at day 4 post DSS colitis induction in A779 (open bars) or saline treated mice (hatched bars) compared to UT group (solid bars). Colon length (panel C) and thickness (panel D) was determined in A779 (open bars) or saline (hatched bars) treated mice compared to UT group (solid bars). Panels E and F represent histological assessment of colitis severity and the % of ulceration in the whole colon section respectively in the groups indicated. Histobars represent means ± SEM for 5–9 mice in each group (9 for UT and 5 for DSS/i.p saline and DSS/A779). Asterisks denote significant difference from UT mice with p<0.05 and # denotes significant difference from DSS/ i.p saline treated group with p<0.05. Panels G and H show representative colon sections from DSS/i.p saline or DSS/A779 treated mice.

To determine the effect of inhibiting the action of endogenous Ang 1–7, the MAS-1 R antagonist A779 was administered (for 4 days) with daily i.p injections at 1 mg/kg dose to DSS treated mice. DSS/i.p saline group did not show weight reduction since DSS treatment was given for 4 days only (Fig 7A). A779 treatment exacerbated the weight loss but did not affect the changes in lymphocytes or neutrophils induced by DSS (Fig 7A and 7B). There was also no effect on colon length or thickness but histologically, there was significant additional deterioration of the mucosal structures and increased ulceration as compared to the DSS/i.p saline group as shown by the examples in Fig 7 panels G and H and quantitatively in panels E and F. At the macroscopic level, A779 treatment resulted in reduction of the DSS induced edema but worsened the erythema, with no effect on the other parameters (Table 4). Prolonged administration of DSS plus A779 to mice (for 5–7 days) resulted in death (data not shown), highlighting the intensified colitis severity as a result of prolonged inhibition of the endogenous levels of Ang 1–7.

**Table 4:** Effect of A779 on macroscopic features of colitis when administered simultaneously with DSS.

% of mice displaying each parameter						
Mice group	Diarrhea	Blood in stool	Adhesion	Erythema	Edema	Ano-rectal bleeding
UT	0	0	0	0	0	0
0.9% Saline + DSS	20	0	100	40	100	0
A-779(1.0 mg/kg) + DSS	20	0	100	60	60*	0

Colitis severity was assessed by macroscopic examination in UT mice, or mice treated with saline or A779 for 4 days along with DSS treatment. Numbers are the means of 5–9 mice for each group (9 for UT and 5 for DSS/i.p saline and DSS/A779).

* denote significant difference from DSS/i.p saline treated mice.

### Effect of Ang 1–7 on phosphorylation of signaling molecules

**Fig 8:**
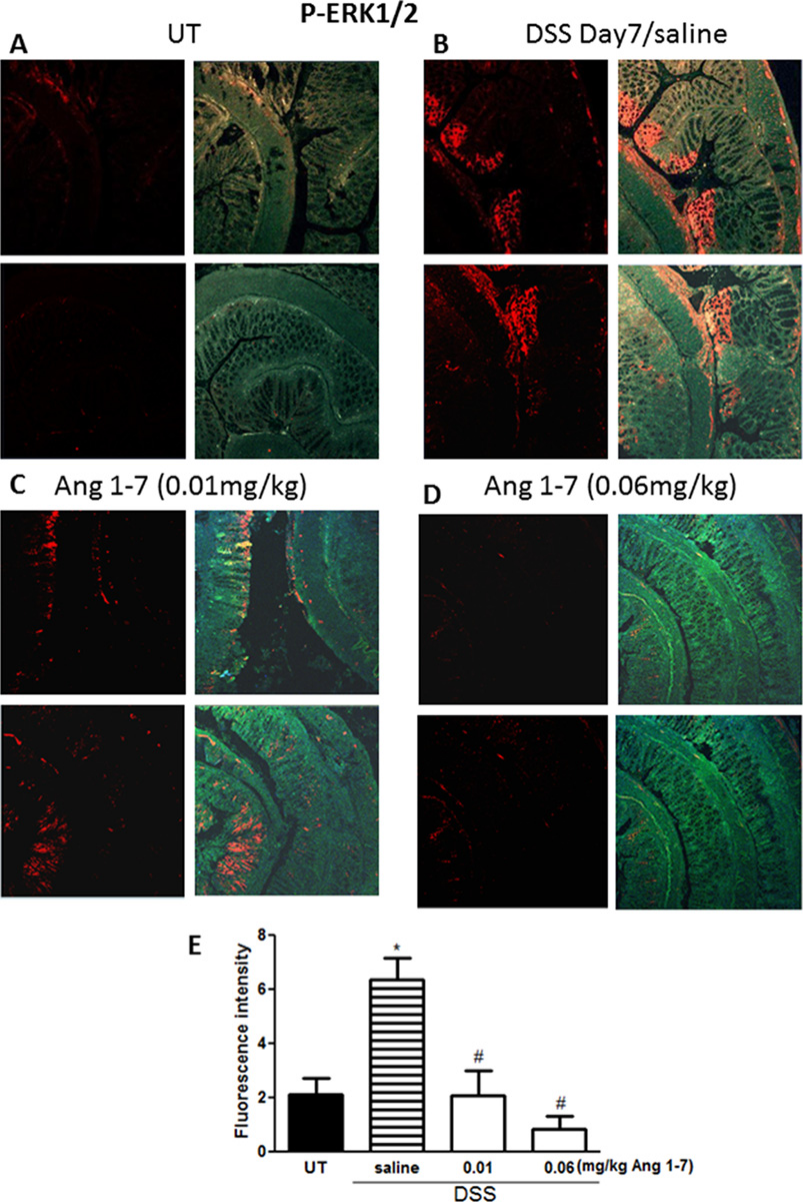
Immunofluorescent detection of phosphorylated ERK 1/2 in colon sections. Colon sections taken from untreated (UT) mice or mice treated with DSS for 7 days plus Ang 1–7 or saline were immunostained with antisera against phosphorylated ERK 1/2. Immunofluorescent (Alexa Fluor) signals shown in left side of panels A-D are overlaid with DAPI stain on right side to show tissue architecture for the conditions indicated. Panel E represents quantitative assessment of fluorescence intensity (arbitrary units). Histobars represent means ± SEM for 3 mice in each group. Asterisk denotes significant difference from UT mice, with p<0.05, and # denotes significant difference from DSS/ i.p saline (vehicle) treated mice, with p<0.05.

**Fig 9:**
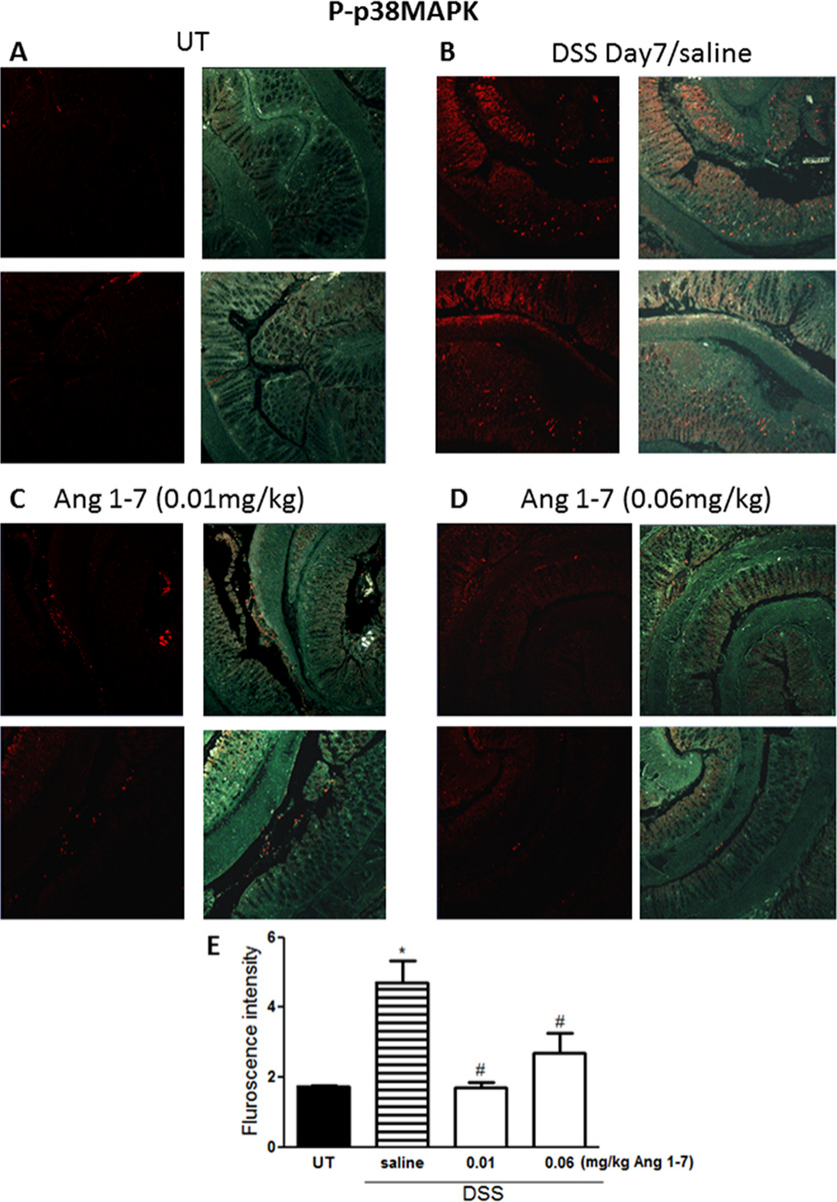
Immunofluorescent detection of phosphorylated p38 MAPK in colon sections. Panel E represents quantitative assessment of fluorescence intensity (arbitrary units). Histobars represent means ± SEM for 3 mice in each group. Asterisk denotes significant difference from UT mice, with p<0.05, and # denotes significant difference from DSS/ i.p saline (vehicle) treated mice, with p<0.05.

**Fig 10:**
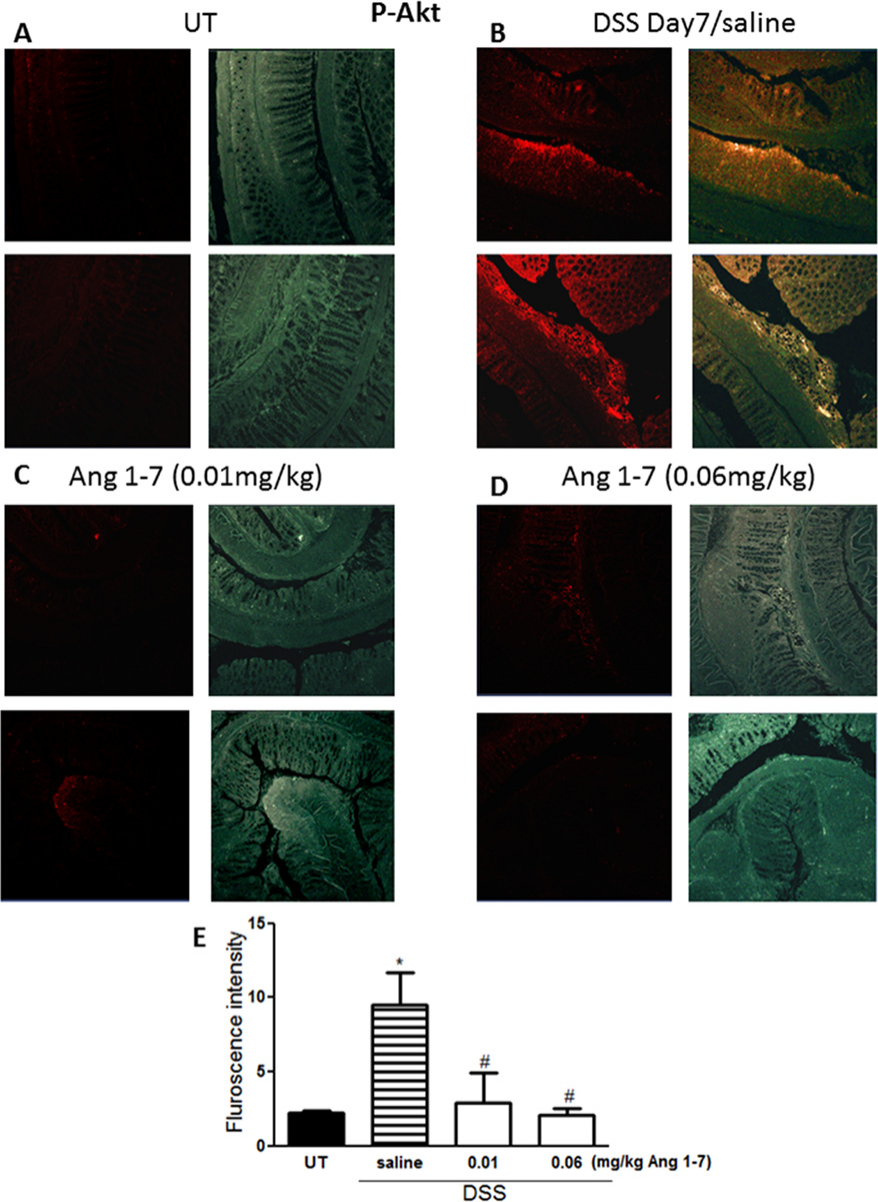
Immunofluorescent detection of phosphorylated Akt in colon sections. Panel E represents quantitative assessment of fluorescence intensity (arbitrary units). Histobars represent means ± SEM for 3 mice in each group. Asterisk denotes significant difference from UT mice, with p<0.05, and # denotes significant difference from DSS/i.p saline (vehicle) treated mice, with p<0.05.

The level of phosphorylated forms of three key signaling intermediates, ERK1/2 (Fig 8), p38 MAPK (Fig 9) and Akt (Fig 10), were measured by immunofluorescence in sections from resected colon tissue of untreated mice or mice treated with DSS (for 7 days) plus daily Ang 1–7 or saline (vehicle) treatment. In each case, expression was enhanced by DSS and reduced by Ang 1–7 back to the basal levels observed in the UT group. In the case of ERK1/2, Ang 1–7 at 0.06 mg/kg dose suppressed phosphorylation even below basal levels (Fig 8E).

### Effect of Ang 1–7 on Ang II levels

**Fig 11:**
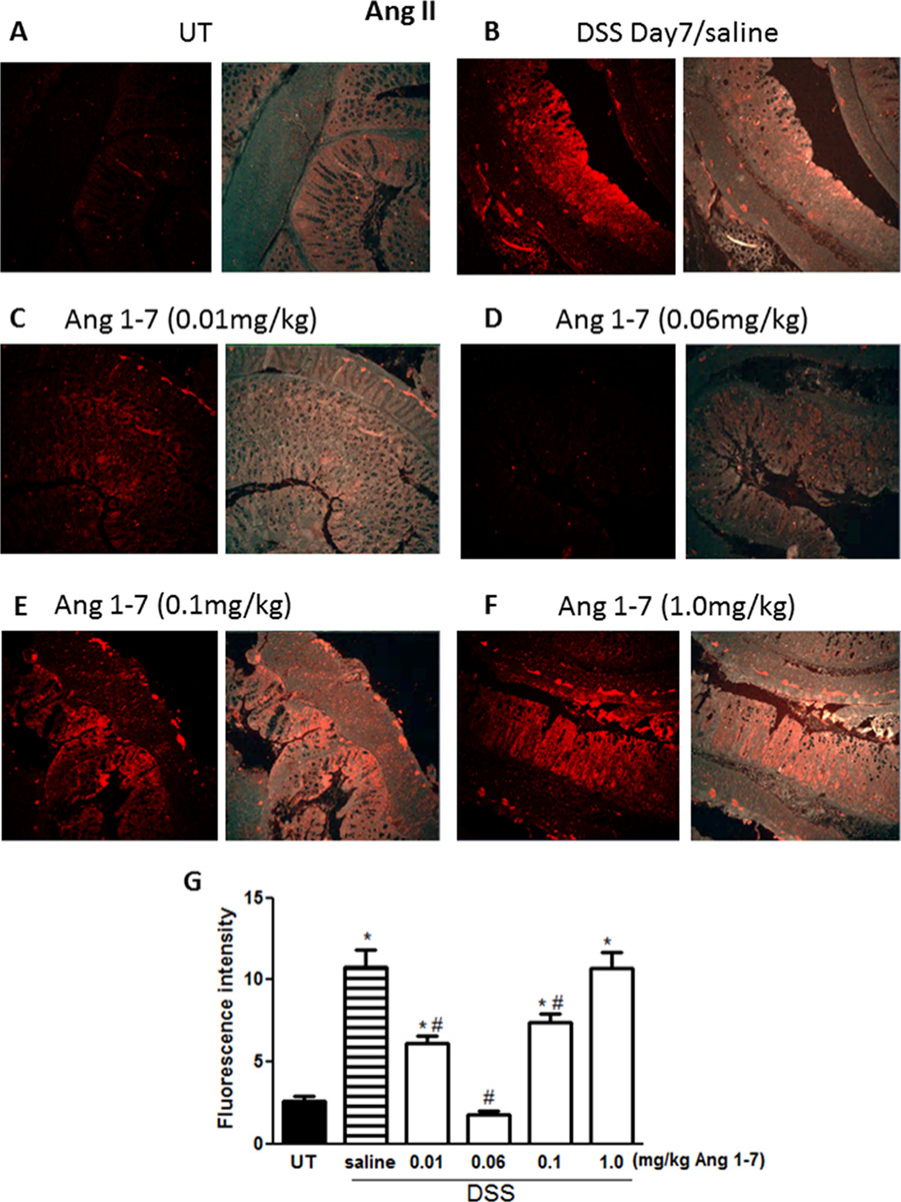
Immunofluorescent detection of Ang II in colon sections. Colon sections taken from untreated (UT) mice or mice treated with DSS for 7 days plus Ang 1–7 or saline were immunostained with antisera against Ang II. Immunofluorescent (Alexa Fluor) signals shown in left side of panels A-F are overlaid with DAPI stain on right side to show tissue architecture for the conditions indicated. Panel G represents quantitative assessment of fluorescence intensity (arbitrary units). Histobars represent means ± SEM for 3 mice in each group. Asterisk denotes significant difference from UT mice, with p<0.05, and # denotes significant difference from DSS/ i.p saline (vehicle) treated mice, with p<0.05.

The expression level of Ang II was examined by immunofluorescence in sections from resected colon tissue of untreated mice (UT) or mice treated with DSS (for 7 days) plus daily Ang 1–7 or saline (vehicle) treatment. Ang II expression was enhanced by DSS administration (Fig 11B) compared to UT mice (Fig 11A). Ang II expression was reduced by daily administration of Ang 1–7 at doses of 0.01–0.1 mg/kg (Fig 11C–11E). In fact, daily administration of Ang 1–7 at 0.06 mg/kg dose (Fig 11D) reduced Ang II to the basal levels observed in the UT group. Daily administration of a high dose of Ang 1–7 (1.0 mg/kg, Fig 11F) resulted in increased Ang II levels similar to levels seen in DSS/i.p saline group.

## Discussion

In this study, we have presented data indicating a novel role of Ang 1–7 in reducing colitis severity in the murine DSS model. Daily i.p injections (at doses of 0.01–0.06 mg/kg) ameliorated colitis severity when given either prophylactically or after colitis induction. Blockade of the MAS-1 R (by A779) aggravated colitis severity, highlighting the involvement of the Ang 1-7/MAS-1 R axis in the pathogenesis of IBD. This protective effect was in part modulated through the expression/activity of several signaling molecules such as Ang II, p38 MAPK, ERK1/2, and Akt.

It has been demonstrated that ACE2 is expressed in the epithelial, sub-mucosal and muscular layers of the colon in both humans and mice [33–35], and its expression levels are increased in the plasma of IBD patients [39]. We observed increased expression of ACE2 and MAS-1 R proteins in the colonic tissues of mice after DSS challenge (Fig 2), and higher Ang 1–7 levels in the inflamed colon homogenates (Fig 1B). Plasma levels of Ang 1–7 were significantly reduced at 7 days post DSS treatment (0.002 ± 0.006 ng/ml) when compared to healthy controls (UT; 0.232 ± 0.134 ng/ml). Partial restoration was achieved with daily i.p injections of Ang 1–7 at 0.06 mg/kg dose (0.061 ± 0.009 ng/ml; Fig 3G). The enhanced expression of the ACE2/Ang 1-7/MAS-1 R axis post DSS challenge might reflect a compensatory protective mechanism to reduce the effect of the inflammatory response. Daily Ang 1–7 administration significantly reduced colitis severity observable at both gross (Table 1) and histological levels (Fig 3E and 3F). In addition, it also opposed the DSS-induced drop in body weight (Fig 3A), and restored the changes in colon length and thickness post colitis induction (Fig 3C and 3D). The protective effect of Ang 1–7 was lost with higher doses *in vivo* (Fig 3). One explanation is that higher concentrations of Ang-1-7 attenuate the MAS-1 R responsiveness through receptor desensitization [40–42]. Another report also showed that Ang 1–7 treated HEK 293T cells at high concentrations redistributed the MAS-1 R to intracellular vesicles and co-localized Ang 1–7 with Rab5 and the adaptor protein complex 2, suggesting regulation by a clathrin-mediated pathway [43].

Enhanced activity of members of the MAPK family, p38 MAPK and ERK1/2 as well as Akt (the downstream target of PI3K), which we observed in colonic sections post DSS treatment (Figs 8–10) has previously been reported in colonic biopsies from IBD patients [44–47]. Treatment with inhibitors of these signaling molecules ameliorated colitis severity in mice and reduced the disease activity index in patients, underlining their role in IBD pathogenesis [44–47]. In this study, we showed that the anti-inflammatory effects of Ang 1–7 treatment were associated with a reduction in the phosphorylated forms of p38 MAPK, ERK1/2 and Akt post DSS induction (Figs 8–10), consistent with previous reports showing reduced activity of these molecules post Ang 1–7 treatment in the anterior pituitary [31], proximal tubular cells [29], and lung tissues in an animal model of lung inflammation [32]. Using immunofluorescence analysis, we observed enhanced colonic expression of Ang II at day 7 post-DSS treatment (Fig 11), confirming western blotting data presented in Fig 2A. Ang II expression was reduced by daily Ang 1–7 treatment (at doses of 0.01–0.1 mg/kg, Fig 11C–11E). This suggest that the anti-inflammatory properties of Ang 1–7 are in part mediated through reduction of Ang II levels.

Regarding the influence of the ACE2/Ang 1-7/MAS-1 R axis in modulating colitis severity, one report demonstrated that administration to mice of GL1001 (as a chemical inhibitor of ACE2) reduced colitis severity [48]. However, it should be noted that the specificity of this inhibitor is questionable, since ACE2 inhibition may increase the levels of other substrates such as Ang I/II, ghrelin, dynorphin, bradykinin, neurotensin and apelin [49]. Other studies (in the murine TNBS model) have demonstrated that ACE2 deficiency results in increased likelihood of developing intestinal inflammation due to the disruption of dietary amino acid homeostasis, innate immunity and gut microbial ecology [50], as well as increased leukocyte recruitment and pro-inflammatory cytokine release in the colonic tissues. These data are consistent with a protective role of the ACE2/Ang1-7/MAS-1 R axis observed in this study.

In regard to the effect of DEX on modulating colitis severity, targeted delivery of DEX to the colon was shown to reduce colitis severity in rodents [51–53]. However, daily i.p administration of DEX was shown to either increase [54], or decrease colitis severity [55, 56]. In our study, we showed that daily i.p injection of DEX at doses 0.01–0.06 mg/kg significantly reduced colitis severity at macroscopic (Table 3) and histological level (Fig 6D–6E).

In conclusion, this study is the first demonstration (to our knowledge) that the Ang 1-7/MAS-1 R axis plays a role in modulating colitis severity, in part through down-regulation of the levels of Ang II and the phosphorylation of key signaling molecules involved in the inflammatory process such as ERK1/2, p38 MAPK and Akt. Blockade of the MAS-1 R inhibited the protective effects of endogenous Ang 1–7 and increased the severity of colitis.
